# Oncotargeting by Vesicular Stomatitis Virus (VSV): Advances in Cancer Therapy

**DOI:** 10.3390/v10020090

**Published:** 2018-02-23

**Authors:** Suman Bishnoi, Ritudhwaj Tiwari, Sharad Gupta, Siddappa N. Byrareddy, Debasis Nayak

**Affiliations:** 1 Discipline of Bioscience and Biomedical Engineering, Indian Institute of Technology Indore, Indore, MP 453 552, India; phd1501171015@iiti.ac.in (S.B.); phd1501171001@iiti.ac.in (R.T.); shgupta@iiti.ac.in (S.G.); 2 Department of Pharmacology and Experimental Neurosciences, University of Nebraska Medical Center, Omaha, NE 68130, USA

**Keywords:** oncolytic virus, vesicular stomatitis virus (VSV), oncotherapy, tumor lysis, apoptosis

## Abstract

Modern oncotherapy approaches are based on inducing controlled apoptosis in tumor cells. Although a number of apoptosis-induction approaches are available, site-specific delivery of therapeutic agents still remain the biggest hurdle in achieving the desired cancer treatment benefit. Additionally, systemic treatment-induced toxicity remains a major limiting factor in chemotherapy. To specifically address drug-accessibility and chemotherapy side effects, oncolytic virotherapy (OV) has emerged as a novel cancer treatment alternative. In OV, recombinant viruses with higher replication capacity and stronger lytic properties are being considered for tumor cell-targeting and subsequent cell lysing. Successful application of OVs lies in achieving strict tumor-specific tropism called oncotropism, which is contingent upon the biophysical interactions of tumor cell surface receptors with viral receptors and subsequent replication of oncolytic viruses in cancer cells. In this direction, few viral vector platforms have been developed and some of these have entered pre-clinical/clinical trials. Among these, the Vesicular stomatitis virus (VSV)-based platform shows high promise, as it is not pathogenic to humans. Further, modern molecular biology techniques such as reverse genetics tools have favorably advanced this field by creating efficient recombinant VSVs for OV; some have entered into clinical trials. In this review, we discuss the current status of VSV based oncotherapy, challenges, and future perspectives regarding its therapeutic applications in the cancer treatment.

## 1. Introduction

Cancer cells can be distinguished physiologically from their normal counterparts for their illimitable replication potential, ability to synthesize their own growth factors, unresponsiveness to the growth-inhibitory signals, and capacity to initiate angiogenesis and metastasis of the tumor. Unlike healthy cells, cancer cells show reduced innate mechanism for detecting and inhibiting virus replication [1]. This allows certain viruses to infect and express lytic genes in cancer cells, resulting in cell death and subsequent cell-to-cell spread of the progeny. Tumor cells support a higher quantity of progeny virus, which subsequently rip through cancer cells in waves of lytic replication before reaching healthy cells, where an intrinsic check and balance mechanism aborts their spread to healthy cells. This tumor-specific lytic property of a virus forms the basis of oncolytic virotherapy. The technical foundation of the concept of using the lytic property of viruses to eradicate cancer cells has existed for a while [2]. Various case reports published earlier mentioned the bystander effect of rabies virus on tumor regression in a patient suffering from cervical carcinoma. Originally intended as a therapeutic intervention against dog-bites, Pasture’s attenuated rabies vaccine showed significant regression of cervical carcinoma in the patients [2]. Following documentation of anti-tumor properties of rabies virus, successively, a diverse set of viruses, including adenovirus (ADV), influenza virus, mumps virus, and Newcastle disease virus (NDV), etc., were tried as therapeutic agents [3]. Since the mid-19th century, clinical case reports citing tumor regression in naturally occurring viral infections is being documented [4]. Some recent clinical case reports emphasized a correlation between viral infection (hepatitis, measles, etc.) with the waning of leukemia [5] and Burkitt’s lymphoma [6,7,8]. Further, to make therapeutic use of tumor regression properties of oncolytic virotherapies (OVs), suitable recombinant viruses were created either by genetic engineering techniques, or by selecting viruses with natural tropism for tumor cells [7]. Though interest in OVs gained momentum, safety concerns associated with virus infection primarily prevented further progress in its therapeutic applications. However, advancements in molecular biology technology combined with virus reverse genetics tools have addressed these issues and brought renewed interest to this field. As a result, genetically modified DNA viruses, such as herpes simplex virus (HSV) and adenoviruses (AdV) were developed as candidate OV agents [9]. The recent documentation of oncolytic properties of Zika virus against glioblastoma stem cells exhibits potential applications in this direction [10]. 

## 2. Vesicular Stomatitis Virus as an Oncolytic Virus

Vesicular stomatitis (VS) virus (VSV) is a prototypic member of the *Vesiculovirus* genus and is placed in the well-defined Rhabdoviridae family. The virus is named as per the classical vesicular lesions in the oral mucosa, dental pad, tongue, lips, nostrils, hooves, and teats of the affected animals. The virus is transmitted by insect vectors and disease is limited to its natural hosts, such as horses, cattle, and pigs [11]. In humans, the infection is mild and asymptomatic. Only accidental cases of human infections have been reported in animal-handlers and laboratory researchers. The wild-type (WT) virus grows to high titers (10^9^ plaque-forming units (pfu)/mL) in a variety of tissue culture cell lines. For these reasons, VSV is used as an excellent biological tool to study basic biological processes and functions, in both in vitro and in vivo systems. Further, various laboratories across the globe has made detailed molecular characterization of the virus, providing useful information and tools for subsequent experimentation.

The viral genome consists of a single-stranded RNA with negative-sense polarity. The 11,161 nt long genome contains five genes that are flanked by 3′-leader and 5′-trailer sequences in the order of 3′-leader–Nucleocapsid protein (N)–Phosphoprotein (P)–Matrix protein (M)–Glycoprotein (G)–Large polymerase protein (L)–trailer-5′ [12]. In the infected cells, the viral matrix protein shuttles between cytoplasm and nucleus and targets host RNA polymerases (RNAP I, II and III) and other host factors resulting in host transcriptional inhibition [13]. It interferes with the nuclear-cytoplasmic transport of nascent messenger RNA (mRNA) by inhibiting Ras-like nuclear guanosine triphosphatase (GTPase) Ran-TC4 activities, and impedes host mRNA export from the nucleus, leading to cytotoxicity [14,15,16]. The matrix protein also interacts with other associated factors such as nucleoporin Nup98 and host Rae1 proteins, which lead to host transcriptional control [16,17]. However, the viral genome translation is unaffected, as *cis-*acting elements present in 5′ end of viral mRNAs promote recruitment of host translational factors. Particularly, the large ribosomal subunit protein rpL40 of host preferentially facilitates cap-dependent translation of viral mRNAs [18]. This mechanism favors comparatively higher viral protein synthesis in the infected host cells [19,20].

**Figure 1:**
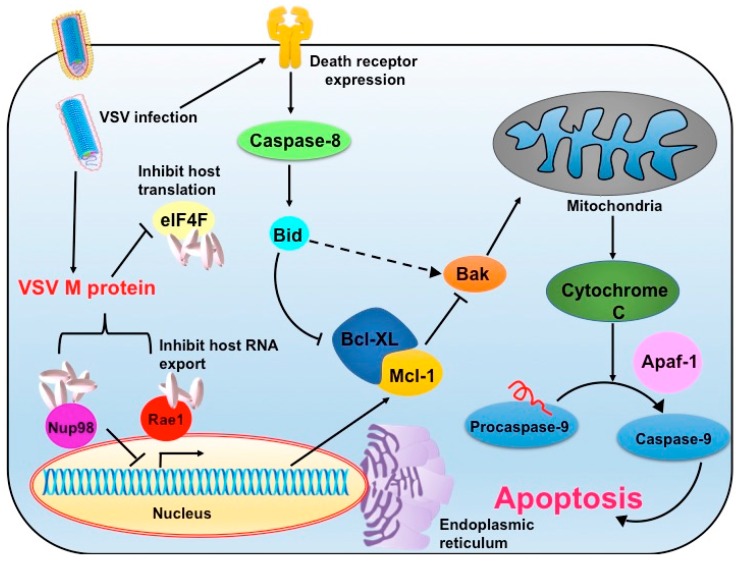
Mechanism of apoptosis induction by Vesicular stomatitis virus (VSV) in the infected cell. The figure depicts possible mechanisms by which VSV could induce apoptosis in infected cells. Depending on the context, either the intrinsic or extrinsic pathway of apoptosis is activated. Mcl-1: Induced myeloid leukemia cell differentiation protein 1; Apaf-1: Apoptotic protease activating factor 1; Bak: BCL2 antagonistic/killer; Bcl-XL: B-cell lymphoma-extra-large; eIF4F: eukaryotic initiation factor 4F; Nup98: nucleoprotein 98; Rae 1: ribonucleic acid export 1.

VSV is a potent and rapid inducer of apoptosis in the infected cells, which is the basis of its oncolytic potential (Figure 1). Cellular pathogenesis in VSV infected cells starts with morphological changes, such as the shutdown of host transcriptional machinery by M protein, which ultimately ends with cell apoptosis [21,22,23]. This early induction of cellular apoptosis is a host defense mechanism designed to limit virus replication and subsequent virus yield. However, the matrix protein modulates overexpression of the host anti-apoptotic protein, B-cell lymphoma 2 (Bcl-2) that allows completion of the viral replication cycle before apoptotic death of the infected cells [24]. The M protein is sufficient to induce apoptosis through the mitochondrial intrinsic pathway by activation of caspase-9, and this is independent of viral replication and de novo synthesis of M protein [25]. By experimenting with a mutant version of M protein that does not activate caspase-9, it became apparent that the mutant VSV could also induce death receptor-mediated (extrinsic pathway) apoptosis, which follows activation of upstream caspase-8 [26,27]. Although M protein inhibits host gene expression and commits the cell to the death cascade, there are other viral components such as viral glycoprotein, which contribute to apoptosis induction. Further, the apoptotic effect of M protein varies with cell types, which allows room to specifically target and lyse the tumor cells [24]. VSV also down regulates and triggers proteasomal degradation of the Bcl-2 family member, the myeloid cell leukemia 1 protein (Mcl-1), a known anti-apoptotic factor that regulates the balance between apoptosis and autophagy. Along with the reduced expression of Mcl-1, caspase-3 activation and cleavage of light chain 3 (LC3) protein decisively contributes to apoptosis [28]. By this mechanism, VSV sensitizes chemotherapy-resistant tumor cells to apoptosis and thus has been postulated to complement chemotherapy regime [28]. As the viral matrix protein alone is sufficient to induce apoptosis, attempts were made to deliver matrix protein to the target cells in the form of plasmid DNA. In this aspect, Zhao’s group has documented that liposome-mediated delivery of plasmid DNA to the nude mice with metastatic ovarian cancer showed desired M expression in target cells. Further, the M expression reversed angiogenesis and tumor metastasis while desired induced apoptosis in the tumor cells [29].

In a different mechanism, VSV is capable of inducing endoplasmic reticulum (ER) stress-mediated apoptosis. The ER is a major organ where a plethora of highly specialized cellular functions involving various macromolecules is orchestrated. Viral protein synthesis and processing (often highly, modified) are extensively ER-associated. As the demand for protein processing increases during viral replication, a complex and adaptive response termed as ER stress is turned on as a cytoprotective mechanism. But, unlike chronic ER stress, transient ER stress is shown to modulate the cellular signalling response particularly the induction of apoptotic caspase-2 induction. Thus, ER stress induction could complement oncolytic potency of rhabdoviruses such as that of VSV which could result in 10,000-fold increase in viral oncolytic activity [30]. Conversely, VSV infection enhances upregulation of Noxa, a Bcl-2 homology domain-containing pro-apoptotic mitochondrial protein whose expression is also increased in ER stress. Thus, Noxa expression in ER stress synergizes and augments cellular apoptosis during VSV infection [31].

Other than directly triggering apoptosis, VSV employs an additional mechanism to cause tumor regression. VSV tends to infect tumor vasculature leading to loss of blood flow to the tumor and subsequent blood-coagulation and massive bystander lysis of neovasculature [29,30]. Infiltrations of peripheral neutrophils to the tumor bed further amplify indirect killing of uninfected tumor cells. Subsequently, infected tumor vasculature shows neutrophil-dependent induction of microclot formation by fibrin deposition within the blood vessels [32]. Collectively, VSV potentiates oncolytic activity with reduced proliferation of malignant cells and enhanced apoptosis of infected tumors [33]. The lack of ionizable oxygen in the tumor microenvironment is a key factor that resists the effect of chemotherapeutic agents and radiation therapy [34]. Hypoxia also induces several adaptive stress responses at cellular level, including inhibition of DNA replication transcription, and protein translation [35]. A hypoxic microenvironment specifically allows the transcription and translation of hypoxia-adaptive genes such as vascular endothelial growth factor (VEGF) and hypoxia-inducible factor 1 (HIF1) [36]. Hypoxic cells show reduced mRNA translation activities compared to normoxic cells. However, VSV overcomes this translational suppression and directs cellular machinery for preferential translation of viral proteins [37]. The VSV has been shown to induce profound cytopathic effects (CPE) and cell lysis in in-vitro hypoxic cell culture and tumor xenograft models [37]. Thus, it can be marked as a potent oncolytic agent to treat established tumors. These intracellular metabolic changes show an opportunity to take advantage to directly target tumor therapy, e.g., a drug that concentrates in hypoxic tissue or toxic gene expression driven by hypoxia-inducible promoters [38]. Together, these studies have demonstrated multiple pathways by which VSV induces cellular apoptosis and hence is considered as a potent oncotherapeutic agent.

## 3. Vesicular Stomatitis Virus and Type I Interferon Signaling

VSV replication is quite sensitive to interferons (IFNs) [39]. Among the three major types of IFNs, the type I interferon (IFN-I) family executes potent anti-VSV activities. The IFN-I family comprises of a dozen IFN-α and one IFN-β subtypes, where both α and β subtypes share the same receptor, IFNAR, for their signal transduction. Upon virus infection, IFNs induce a broad range of gene expression, which not only interferes with virus replication but also modulate host adaptive immune responses. Various in vitro and in vivo studies have corroborated that phosphorylation of double-stranded RNA-dependent protein kinase (PRK) coupled with IFN-I signaling impedes VSV replication [40]. Both viral gene expression and infectious particle release are affected by pre-treatment of cells with IFN-β [41]. Therefore, the intrinsic nature of cancer cells having lower or defective IFN-I signaling makes them more susceptible to VSV-medicated oncolysis [42]. For example, human bladder cancer cells with lower IFNAR expression are susceptible enough for VSV-oncolysis [43] compared to head and neck cancer cells expressing higher α2a and IFN-β subtypes, and are therefore nonresponsive to VSV-induced oncolysis [44]. In immunocompetent hosts, defects in protein kinase R (PKR) and its downstream signaling pathways including alteration in IFN signaling results in synergetic action of caspase-dependent apoptotic activity by VSV [45]. Furthermore, VSV replicates faster in cells having defects in tumor suppressor genes such as p53 [45]. VSV induces faster apoptosis in transformed cells having mutations in myelocytomatosis (*Myc*), Rat sarcoma (*Ras*), or tumor protein 53 (*p53*) genes, as well as tumors overexpressing Bcl-2 genes that are associated with uncontrolled cell division, metastasis, and angiogenesis [45]. Importantly, the vast majority of tumors have activated Ras signaling pathways that facilitate VSV-mediated oncolysis [46]. Overall, by exploiting the knowledge of genetic defects in tumors, remarkable progress in VSV-oncotherapy research has been done by creating designer VSVs fitting to the tumor milieu. 

## 4. Advantages and Limitations of Vesicular Stomatitis Virus as Oncolytic Virotherapy

To induce potent tumoricidal activity, it is desirable to have OVs with higher replication kinetics so that higher viral yield can be achieved in a short time. This facilitates faster spread of virus in the tumor and host immune system to have less time to induce systemic anti-viral responses. The major limitations in therapeutic applications of OVs are their limited replication efficiency and unwanted spread to the surrounding healthy tissues. Compared to other OVs, VSV has a shorter replication cycle, where assembly of first batch of progeny virions is around the same time as the secondary viral genome transcription (2–3 h post infection). VSV neither causes any cell-transformation, nor any immune-mediated pathogenesis in the host. Further, the viral envelop glycoprotein (G) has broad tissue tropism and hence been adopted for the establishment of numerous pseudotype viruses [47]. Because of all these advantages, VSV could be targeted to a variety of tumor cells in vivo. However, when effective tumor regression requires multi-dose administration of OVs, sometimes choosing VSV as oncolytic agent could be disadvantageous. Particularly in the immune competent host, multi-dose applications of VSV prompt the adaptive immune response, primarily by eliciting a humoral immune response against the G protein, which restricts the active period for oncolysis. The humoral response could result in the generation of G-neutralizing antibodies in the host that may cause virus sequestration in organs, mainly in the liver and spleen. The systemic administration of the virus may result in its transduction to antigen presenting cells (APCs), which activates downstream immune (B cell) responses with increased production of neutralizing antibodies; thereby causing rejection of subsequent administered VSV. However, this problem can be overcome by masking the viral envelope or by changing certain amino acid residues of G. The detailed strategy for G modification is discussed later. Another disadvantage of VSV is due to its neurotropic nature. Neurotropism is primarily mediated by G and partly by the M protein. In experimental animal models, VSV preferentially grows in the olfactory lobe and cerebellum, causing fatal viral encephalitis [48]. However, these concerns are already addressed by making alterations in the viral M and G protein, generating safer and more efficacious recombinant VSVs. Taken together, VSV, being a non-human animal virus with a potent lytic activity, is being proved as a potential OV agent. Further, combined with its tumor-selective replication properties aided by shorter lifespan, it is an attractive OV candidate with recombinants having entered clinical trials.

## 5. Amelioration of Vesicular Stomatitis Virus-Associated Neuropathogenesis: Different Approaches

### 5.1. MicroRNA Targeting

The host often produces microRNAs (miRNAs) that exacerbate viral pathogenesis in a tissue-specific manner. For example, cellular miRNA-125 is highly upregulated in brain cells during central nervous system (CNS) viral infection, which is known to modulate innate immune responses that could trigger immune-mediated neuropathogenesis. The best approach to counteract a specific miRNA is the generation of a complementary RNA (cRNA) sequence against said miRNA, where a viral gene delivery approach is used. In this approach, Kelly et al. created an attenuated rVSV by incorporating miR-125-specific cRNA sequences in the 3′ UTR region of the viral genome which showed significantly lower neuropathogenesis [49]. In addition to reduction of toxicity, miRNA strategies could be used for oncotargeting. Here, preferential tumor lysis could be achieved by taking advantage of differential miRNA or cellular protease expression patterns in the tumor environment. For example, let-7 miRNA show low expression in cancer cells [50]. By adding a complementary RNA sequence of let-7 microRNA at 3′ end of M gene of VSV, Edge et al. demonstrated that this recombinant VSV preferred to express matrix protein at a higher rate in low let-7 microRNA expressing cancer cells leading to cellular apoptosis [51]. 

### 5.2. Modifications of M

**Figure 2:**
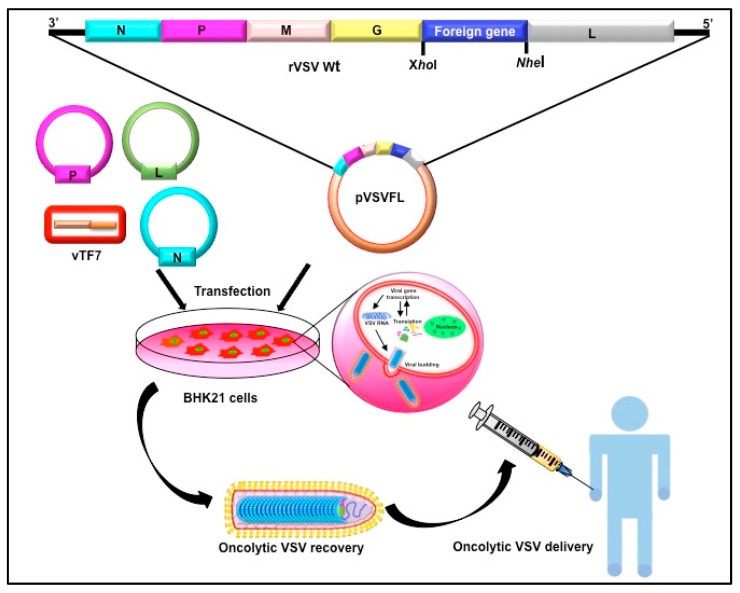
Reverse genetics use in making customized VSV targeting tumor lysis. Schematic presentation of VSV reverses genetics system. Plasmid DNA harboring full-length viral genome and other accessory proteins are transfected to the supporting cell line to initiate virus genome transcription and replication. A helper virus or another plasmid expressing T7 polymerase is required to initiate transcription. Later, the accessory proteins (N, P, and L) takeover viral genome replication and transcription leading to the recovery of new viruses. pVSVFL: plasmid containing cDNA copy of VSV full-length genome.

**Figure 3:**
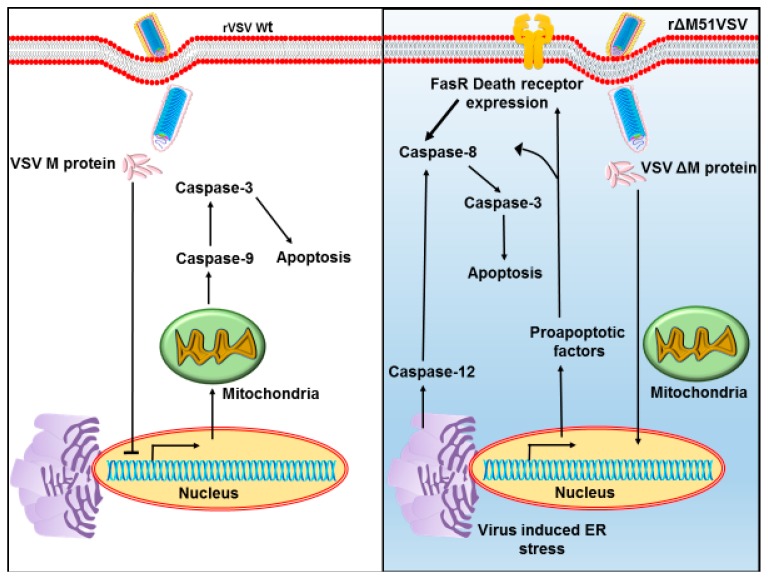
Modified matrix protein is a superior oncolytic agent. The left panel shows the action of wild-type matrix protein and pathways leading to cellular apoptosis. The right panel depicts mutant of matrix protein efficient in apoptosis in cancer cells. ER: endoplasmic reticulum; rVSVwt: recombinant VSV wild type; rΔM51VSV: recombinant VSV with mutation at 51st position in its matrix protein.

VSV reverse genetics systems are well established and many advances have been done to precisely edit the viral genome (Figure 2). To ameliorate neuropathogenicity, appropriate genome modifications efforts were made to create recombinant viruses (rVSVs) with desirable oncolytic activities [52]. Initially, the matrix protein was mutated. The ΔM51 mutant created by deletion of methionine at 51st position (VSVΔM51) showed reduced cytotoxicity and was found to be ineffective in blocking IFNs (IFN-α) expression in the infected cells [53]. Substitution of methionine to arginine at the same position (rM51R-M) did not inhibit host cell gene expression, but retained replication and apoptosis activities of the WT virus. Hence, the rM51R-M virus was regarded as a superior oncolytic agent for its ability to induce IFN-I gene expressions and antiviral responses in healthy cells and it was therefore was chosen to target tumor cells having defective IFN-I signaling (Figure 3). As an alternate approach, translational regulation of the M was targeted by incorporation of picornaviral internal ribosome entry sites (IRES) upstream of the M coding region. Here, IRES sequences of human rhinovirus type 2 (HRV2) and foot and mouth disease virus (FMDV) were individually inserted at the upstream of the M start codon to suppress M expression. Subsequent neuroattenuation was achieved with the rVSVs [54]. 

### 5.3. Modification of G

To counter neurotoxic and immunostimulatory effects associated with the G protein, a mutational approach for G modification has been explored. Various point mutations in G were created (G5, G6, and G6R). The resultant G mutants did not inhibit IFN-I expression like that of M mutants and also did not interfere with host mRNA transcription and translation machinery. However, the cytopathic effect of G mutants experimented in B16 and MC57 tumor cells were comparable to that of M mutant or WT virus [55]. In another approach, deletion mutants were created by the deletion of cytoplasmic domains of G from the 29th amino acids (aa) to the 9th or 1st aa (VSV-CT9 or VSV-CT1). Interestingly, these deletion mutants were not only attenuated, but more importantly, showed less immunogenicity in eliciting a humoral immune response [56]. Moving further in this direction, complete deletion of G from the viral genome and rescuing the mutants (VSV-ΔG) in G expressing cells resulted in single cycle replicating virions. These virions showed effective oncolysis of gliomas with minimal toxic effects to neurons as compared to wtVSV, and were therefore experimentally used as an adjuvant to surgical treatment of high-grade gliomas [57]. As these tools were readily available, various preclinical studies were started in a variety of malignancies, such as breast cancer, liver cancer [58], and colorectal cancer [51] models, which showed encouraging results. 

To further counter the issue related to G-antibody mediated viral neutralization, chimeric VSVs were created by replacing its glycoprotein with that of an Arenavirus (lymphocytic choriomeningitis virus, LCMV); the resultant (rVSV-GP) showed neuroattenuation while simultaneously escaping the humoral immune response [59]. Additionally, VSV pseudotyped with the envelope glycoprotein of non-neurotropic LCMV (LCMV-GP3VSV-GP) showed little infectivity for primary human and rat neurons in vitro and in vivo, but enhanced infectivity for brain cancer cells, exhibiting a wide therapeutic window for clinical applications against malignant gliomas [60]. In another approach to counter immune rejection associated with G neutralizing antibodies, genome level glycoprotein exchange between serotypes of VSVs was proposed. Given that VSVs occurs in two major serotypes, i.e., Indiana and New Jersey, the G protein switching could solve this issue to some extent. Viral envelope protein epitope-shielding with polymer coats (poly [*N*-(2-hydroxypropyl) methacrylamide] (Phpma) bearing reactive 4-nitrophenyl esters on pendent diglycyl side chains) is also proposed to avoid immune rejection [2]. Similarly, by covalent modification of G with polyethylene glycol (PEG) or function-spacer-lipid (FSL)–PEG, antibody neutralization and virus sequestration issues could be avoided [61]. 

### 5.4. Modulating Viral Replication

In an entirely different approach to reduce neurotoxicity and attenuate viral pathogenesis, a semi-replicative VSV vector (srVSV) system was created where genes essential for VSV genome replication and transcription were placed in two sets of viral vectors. Each subgenomic vector contained a partial viral genome, thus requiring both vector combinations to complete viral genome replication in infected cells. In this system, combinations of the VSVΔG vector with either of the two trans-complementing deletion mutants of VSV polymerase L (VSVΔL) or phosphoprotein P (VSVΔP) or the mutual combination of VSVΔP/VSVΔL vectors were co-administrated. The resultant vector replication was proven to be safe in the host, with only a marginal reduction in its antitumor efficacy [62]. The co-propagation of srVSV systems, especially the VSVΔG/VSVΔL, demonstrated higher lytic property inhuman glioblastoma cell lines and subcutaneous xenografts with no recombination or reversion into replication competent virus. In contrast to WT virus, injection of VSVΔG/VSVΔL into the brain triggered long-term regression of tumors and did not show neurotoxicity [62]. In separate approaches, shuffling the position of individual genes in the viral genome and introducing neutral genes such as green fluorescence protein (GFP) were also attempted [63]. The resultant gene re-shuffled VSV-ΔG-GFP exhibited altered protein expression ratios that contributed to reduction in viral replication cycles and made the virion attenuated [64]. All these modifications demonstrate that the viral genome is highly amenable, and neurotoxic proteins such as G and M could be suitably modified to attain a desired level of safety while preserving its oncolytic potentials. 

## 6. Advancement in the Tumor-Specific Targeting of Vesicular Stomatitis Virus

Ideally, an oncolytic virus should have the following properties for a better therapeutic manifestation: (i) specificity in targeting tumor tissue; (ii) ability to extravasate tumor tissue; (iii) escape antibody neutralization; and (iv) lack of neurotoxicity. As the issues related to neurotoxicity and immune neutralization have been addressed, the next set of modification was made to achieve higher tumor lysing efficacy. To achieve this, customized rVSVs were engineered primarily through three modes. In the first mode, the viral entry to the tumor cell was controlled by the necessary glycoprotein G modifications. In the second mode; oncotargeting was achieved by preferential viral replication in the tumor cells. Here, viral genes (M) whose expression counters the cellular antiviral responses (double-stranded RNA and IFN response) are mutated, so that a certain level of the antiviral state is maintained in the healthy cells, while the cancer cells being defective in innate response became more susceptible to viral replication. In the third mode, oncolytic activity was enhanced by co-administration of rVSV with suitable bioreagents such as complementing with cytokine or monoclonal antibodies. Here synergistic effect of apoptosis of both reagents resulted in higher oncolytic activity with enhanced specificity. 

Classically, tumor directing is achieved by displaying tumor-targeting ligands on the virion surface, where the viral glycoprotein is genetically altered to bind specific tumor receptors at higher affinity. Here foreign peptides are genetically inserted into VSV-G to enhance tumor tropism [65,66]. Stephen Russel’s group has demonstrated that both small (cyclic arginine-glycine-aspartic acid; cRGD, 9 aa) and medium-sized (echistatin, 49 aa) insertions in the specific position of G could produce infectious VSVs with higher tumor vasculature-targeting ability [67]. For example, cyclic Arg-Gly-Asp (cRGD) has a higher affinity to bind to cell surface integrins which is critical for tumor initiation, progression, and metastasis [68]. Hence the cRGD-integrin targeting and subsequent blocking integrin function is an attractive strategy for cancer therapy. Following this concept, genetic modification conducted in few viruses (e.g., parvoviruses, adenoviruses, and measles virus) resulted in displaying the cRGD motif on the virion surface with a stronger affinity for tumor cells [69]. 

Unlike the above-mentioned strategy of partial modification of G, by replacing entire G with a foreign virus glycoprotein, desired tumor targeting was achieved. For instance, VSV pseudotyped with Sindbis virus glycoprotein resulted in higher affinity for human epidermal growth factor receptor protein, human epidermal growth factor receptor 2 (Her2/neu) overexpressing breast cancer cells. In another case, VSV-G was replaced with a chimeric Sindbis virus glycoprotein modified with insertion of a synthetic immunoglobulin G (IgG) Fc-binding domain against *Staphylococcus aureus* protein A [70]. The resultant infectious pseudotype VSV (called as ZZ-modified) showed enhanced preferential targeting of Her2/neu-expressing breast cancer cells. Further, in the presence of Her2/neu monoclonal antibodies, these recombinant replicating VSVs were specifically targeted to breast cancer cells and showed cell-specific oncolytic activities [66,67]. Taking a step forward, Gao et al. have implanted Her2/neu overexpressing mouse mammary tumor cells in the Balb/c mouse and measured tumor lytic activity of this chimeric VSV [71]. By modulating host immune response by injecting monoclonal antibodies directed against a negative regulator of T-cell activation, the cytotoxic T-lymphocyte antigen-4 (CTL-4) their group demonstrated higher therapeutic efficacy of this recombinant virus against implanted tumors in Balb/c mice. Here, cytotoxic cluster differentiation 4 (CD4) T cells gained anti-tumor activities against multiple epitopes of the tumor and in synergy with the lytic function of VSV, resulted in higher tumor clearance [72]. When these CD4 T cells were transferred from a cured donor host to the recipients with Her2/neu expressing tumors, these anti-tumor CD4 T cells independently facilitated tumor regression in the recipient host. The tumor clearance was accompanied by the higher expression immuno-modulating cytokines such as IFN-γ, interleukin (IL)-4, and IL-17 cytokines in the transferred T cells [72]. 

Customized recombinant VSVs were also made to target the p53 gene functions, the master regulator of diverse cellular processes. The p53 is a tumor suppressor protein that inhibits tumor development by modulating cell cycle signaling pathways leading to DNA repair, senescence, apoptosis, or activation of innate immune pathways [73]. Taking these into consideration, two p53-recombinant VSV vectors were created, one with murine p53 (VSV-mp53) and the other without a functional M protein (VSV-ΔM-mp53). Both the recombinants induced a high level of p53 expression and retained oncolytic activities. However, the VSV-ΔM-mp53 was enormously attenuated in vivo as it allowed expression of host cytokine IFN-I [74]. A single dose inoculation of VSV-ΔM-mp53 in immunocompetent mice with ectopic metastatic mammary adenocarcinoma showed tumor clearance with increased host survival and hence regarded as a better candidate [74]. 

Taking advantages of tumor expressing antigens, oncotargeting recombinant viruses were made with higher affinity for such receptors. In this method, CD133 or prominin 1, a highly expressing tumor antigen found in glioma, human hepatocellular carcinoma (HCC), in addition to the cancer stem cells (CSCs), was targeted for OVs. Earlier, it was reported that the measles virus (MV) glycoprotein modified with incorporation of a single chain antibody fragment (scFv) could enhance antibody mediated cell fusion [75]. Taking one step further in this direction, a chimeric recombinant VSV was created where VSV-G was replaced with a modified glycoprotein of MV, which showed higher affinity to CD133. This recombinant exhibited enhanced tumor-selective and tumor lytic activities in an orthotropic glioma model of mice [76].

### 6.1. Increasing Tumor Lysing Efficacy

To enhance tumor lytic activity, strategies were made to create rVSVs expressing pro-apoptotic, immunomodulatory, or suicide cassettes [41,71]. In this direction, Woo et al. made chimeric VSV with equine herpes virus-1 glycoprotein G (gG), which is a viral chemokine binding protein (vCKBPs). The viral vCKBPs were known to bind chemokines subfamilies of C, CC and CXC, and block immune cell infiltration to the inflammation site. Although immune cell infiltration is desired for host protective action, often, in the case of oncolytic virotherapy, infiltration and subsequent anti-viral action of immune cells, particularly natural killer (NK) and NK T cell migration to virus-replication sites, results in quick viral clearance and negatively impacts tumor lysis. By making rVSV with secreted form of vCKBPs, Woo’s group has shown that NK and NK T cell migration to virus-replication sites were modulated (reduced), which led to higher tumor necrosis with increased survival of experimental animals [77]. In a similar concept, the murine gammaherpesvirus-68 broad-spectrum and high-affinity chemokine-binding protein (M3), a known immune modulator that suppresses cellular inflammatory responses, was introduced to the rVSV-MΔ51 backbone. The resultant recombinant showed diminished inflammatory responses as compared to VSV-MΔ51. Reduced neutrophil and NK cell infiltration in to the lesion site was observed in the orthotropic implanted rat model of human hepatocellular carcinoma (HCC) [78]. Similarly, the same group also engineered rVSV-UL141 armed with a protein of human cytomegalovirus (HCMV) known to down regulate the CD155 ligand that activates NK cell migration. This recombinant virus exhibited reduced recruitment and intra-tumoral accumulation of NK and NK T cells that favored tumor clearance in a rat model of HCC with limited toxic effect on the host [77]. Another approach was shown to facilitate tumor extravasation of viruses and further spread of progeny viruses through the tumor cells to enhance tumor clearance. In this context, a rVSV designed to express membrane fusion (F) protein of paramyxoviruses (L289A, mutant fusion protein derived from Newcastle disease virus) was demonstrated to induce syncytia formation among the tumor cells at neutral pH [79]. In vivo administration of this recombinant exhibited syncytia formation with enhanced cytotoxic effects against multi-focal HCC in the livers of immune-competent rats with no collateral damage to the hepatic parenchyma surrounding the tumor [79]. Further, this fusogenic VSV showed significant host survival rates as compared to the non-fusogenic control virus in the treated animals, suggesting its higher therapeutic index values. Similar to paramyxovirus F protein, incorporation of reovirus fusion-associated small transmembrane (FAST) in the VSV-ΔM51 backbone resulted in improved oncolytic activities [80].

### 6.2. Oncolytic Vesicular Stomatitis Viruses Expressing a Suicide Gene

VSV can be armed with suicide gene cassettes that can greatly enhance its tumor-killing properties. For example, herpes virus thymidine kinase (TK) serves as a suicide cassette. HSV-TK phosphorylates the nontoxic prodrug ganciclovir (GCV), which is incorporated into cellular DNA and inhibits its replication, leading to apoptosis. In this notion, rVSV-expressing HSV-TK showed enhanced oncolytic activity [81]. Intratumoral inoculation of this rVSV further stimulated antitumor cytotoxic T-cell activity, a critical factor for tumor cell clearance. It has also been reported that the TK/GCV system has further benefits. The TK expressed from one cell can directly trespass to the adjacent cells, thereby enhancing tumor killing through a “bystander effect” [82]. Other than the TK, the fusion protein comprising the suicide gene of *Escherichia coli*, cytosine deaminase (CD) in conjunction with bacterial uracil phosphoribosyltransferase (UPRT) was introduced to VSV to make recombinants (VSV-C:U) [83]. The mammalian cells do not express CD, while it is present in bacteria and fungi, where it is responsible for deaminating cytosine to from uracil. CD can also deaminate 5-fluorocytosine (5-FC) to a potent chemotherapeutic agent, 5-fluorouracil monophosphate (5-FU). UPRT is a key enzyme in salvaging pyrimidine where it catalyzes transfer of a ribosyl-phosphate group to uracil, resulting in the formation of uridine-monophosphate (UMP). It can also convert 5-FU to 5-FUMP. Downstream, the concomitant action of both enzymes produces toxic metabolites, which incorporate into both DNA and RNA of host cells, resulting in cell death. The VSV recombinant expressing CD/UPRT cassettes exhibited normal growth properties, along with higher expression of biologically active CD/UPRT that resulted in the local production of 5-FU from the systemic administered 5-FC in target cells. In Balb/c mice, it showed considerable reductions in the malignant growth of syngeneic lymphoma (A20) or mammary carcinoma (TSA), and also exhibited extensive bystander effect, as compared to treatment with rVSV or with control 5-FU alone. This study also demonstrated that IFN-γ-secreting cytotoxic T cells were activated by tumors upon VSV-C:U treatment and resulted in higher tumor regression [83]. Moving further in this direction, CD:UPRT genes were introduced to the VSV-MΔ51 platform [84].The recombinant VSV-MΔ51 expressing CD:UPRT triggered a stronger interferon response compared to VSV-MΔ51, thus allowing and restricting the virus to replicate and lyse the target tumor cells [84]. The list of recombinant VSVs expressing suicide genes is depicted in Table 1. 

## 7. Next Generation Vesicular Stomatitis Virus as Oncolytic Virotherapy: Immunomodulatory Function 

The synergistic relationship between immunotherapy and oncolytic virotherapy seems encouraging and deserves more exploration. VSV recombinants were engineered to express either singly or in combination with multiple suicide cassettes, enzymes, ion channels modifiers, immune-stimulatory cytokines, etc. [85]. Taking advantage of immune-stimulatory cytokines known to potentiate tumor clearance when administered as gene therapy, the rVSVs were generated with higher oncolytic activities. For example, IL-4 directs regression of malignancies such as melanoma, glioma, and colon carcinoma [86]. Tumor regression by IL-4 is exerted by augmenting antitumor effector T cell responses supported by host antigen presenting cells (e.g., granulocyte and macrophages) as well as the direct anti-proliferative action of IL-4 [87]. In this line, the recombinant VSV expressing IL-4 (rVSV-IL4) exhibited considerably higher oncolytic activity against breast cancer or melanoma tumors in murine models [81]. Recombinant VSVs expressing proinflammatory cytokines such as IL-12 (VSV-IL12) showed direct cytotoxic effects on murine squamous cell carcinoma of the head and neck. In combination with costimulatory agents such as granulocyte-macrophage-colony-stimulating factor 1 (GM-CSF1), it showed a synergetic effect against tumor regression [88]. The underlining mechanism lies in the immune-stimulatory effect of GM-GSF, known to enhance the recruitment of antigen presenting cells along with augmented CD8 T-cell response [89]. In this context, recombinant VSV-expressing mouse GM-CSF was explored to target Her2/neu expressing breast cancer cells [90]. As expected, the synergetic effect of co-stimulation and viral lytic activity resulted in faster elimination of Her2/neu-expressing peritoneal implants (D2F2/E2 cells) with elevated anti-tumor T-cell effector response in the murine host [90]. On a different note, recombinant VSV-expressing proinflammatory cytokines, interleukin 23 (IL-23) (VSV23) was created. IL-23 has antitumor, antimetastatic activity, and is an inducer of TNF-α. Although the resultant VSV-23 showed higher oncolytic potency, it was attenuated particularly in CNS due to enhanced nitric oxide (NO) response characterized by reduced viral replication and mortality [91]. Similarly, rVSV-expressing interleukin28 (IL-28) showed effective antitumor activities both in vitro and in vivo studies [92]. Intra-tumoral administration of rVSV expressing IL-28 sensitized tumor cells to NK cell recognition and activation, revealing a new understanding of immune-virological relation for exploitation by OV in immune competent hosts [92]. 

Another important milestone in moving towards clinical application has directly stemmed from the creation of recombinant VSV expressing human interferon-β (VSV-IFNβ) [93]. This is the third IFNβ expressing recombinant virus of the *Mononegavirales* order approved for the clinical trial. To evaluate safety and maximum tolerable dose (MTD) of VSV-IFNβ, the phase I clinical trial was conducted with a modified recombinant expressing sodium iodine symporter (NIS) along with IFNβ (VSV-IFNβ-NIS) in the patients with refractory solid tumors (NCT02923466). Recently, this recombinant also entered more phase I clinical trials aimed at treating patients with stage IV or recurrent endometrial cancers, as well as relapsed or refractory multiple myeloma (NCT03120624), acute myeloid leukemia, or T-cell lymphoma (NCT03017820), and patients with drug-resistant solid tumors (NCT01628640). Safety studies of VSV-IFNβ-NIS have shown encouraging results in the natural host model (pig). Seroepidemiological studies conducted to document pathogenicity and transmissibility of the virus in non-infected animals following systemic administration of virus in different animals in close contact with treated groups showed the virus to be non-pathogenic and non-transmissible in the herd [94]. Further safety studies done in C57BL/6 mice showed no apparent neurovirulence or any visible pathogenesis at very high dosage (5 × 10^10^ TCID50/kg; TCID50: 50% tissue culture infectivity dose) of systemic administration [95]. Going forward, the most interesting studies conducted in pet dogs showed positive outcomes in intravenous treatment of VSV-IFNβ-NIS and specifically addressed the feasibility and tolerability issues. It is well appreciated that canines develop cancer spontaneously like that of humans. In this study, using clinical relevant models, LeBlanc group tested the efficacy of VSV-IFNβ-NIS in a group of 11 pet dogs with advanced or metastatic cancers. Remarkably, all canine patients showed measurable regression of cancer while no shedding of infectious viruses were observed [96]. Taken together, these studies support further clinical trial of VSV-IFNβ recombinant in human patients.

Ongoing efforts to improve safety and efficacy of rVSVs are bringing newer and better OV candidates. For example, a previously described VSV-ΔM51 platform was chosen for incorporation of immune genes. Stephenson et al. introduced a secreted form of optimized human IL-15 (opt.hIL-15), (VSV-opt.hIL-15) that enhanced the induction of both NK and T-cell responses [60]. The localized expression of IL-15 by VSV in the tumor microenvironment induced anti-tumor CD8 T-cell responses in a murine model of colon carcinoma with enhanced survival of treated mice as compared to the control group. However, systemic administration of exogenous IL-15 failed to induce CD8 T cell response. In a similar line, a co-stimulatory molecule, CD40 ligand expressing VSV was generated (VSV-CD40L), and it showed a significant increase in T cell priming with augmented immunotherapeutic properties along with conventional oncolytic activities [97]. A list of recombinant VSV expressing immunomodulatory genes is presented in Table 1. 

### Working with Immune System for Oncolytic Virotherapy Efficacy

For higher efficacy, OVs should escape from immune-mediated rejection. It is well documented that components of the innate immune response such as myeloid differentiation primary response gene 88(MyD88) signaling is essential for the oncolytic activity of VSV [98]. MyD88 plays a major role in innate sensing of the virus through Toll-like receptors (TLRs)–ligand engagement, and could also initiate MyD88-dependent expression of interferons (α, β), which exert anti-viral immune responses. Contrary to the notion that effective anti-viral innate response negates oncolytic activity of viruses, Wongthida et al. demonstrated that MyD88 signaling is critical for VSV oncolytic activity [98]. They argued that the lack of MyD88 results in lesser production of proinflammatory cytokines and decrease infiltration of tumor-killing neutrophils. MyD88 is also pro-IFN-producing and it absence could result in the decrease of IFN-I production in healthy cells, and thereby making these cells susceptible to virus lysis. Thus the idea to have intact innate immune response in the host is necessary for target viral replication and intensification of antitumor effects [99]. Considering the role of immune response in virus induced tumor clearance, the right strategy for OV should be designed in a manner that allows activation of the innate immune response after the replication of virus to an extent needed to induce antitumor effect to improve the efficacy of virotherapy. To avoid immune rejections, the OVs could be administered with carrier cell lines that mask viral antigen. Hence, an established syngeneic cell line could be used as a Trojan horse to transiently sequester viral antigen during systemic delivery. As a proof of concept, CT26 murine colon carcinoma cells infected with VSV effectively shielded it from neutralizing antibodies during systemic administration in murine hosts [100]. Further, VSV can be engineered to express tumor-associated antigens to induce adaptive T cell responses directed toward achieving potent cytotoxic T cell functions for systemic therapy against metastatic tumors. It has been demonstrated that CD8^+^ T cell activation against tumor-associated epitopes has enhanced viral oncolytic activities by generating a tumor-specific adaptive immune response [101]. Intra-tumoral virotherapy using rVSV engineered to express tumor epitopes in the presence of circulating tumor antigen specific T cells showed better results in tumor clearance [101]. Thus, combination of adoptive T cell therapy along with virotherapy was shown to enhance systemic tumor regression. As research in molecular immunology is advancing, previously unnoticed factors that could augment apoptosis are also being unraveled. For example, cancer cells showing higher activation of nuclear factor erythroid 2-related factor 2 (Nrf2), a transcriptional regulator, is shown to be vulnerable to VSV-induced apoptosis. In this context, using VSV-ΔM51 platform, Lin’s group has documented that Nrf2 signaling impairs IFN-I in Nrf2 overexpressing cells; thereby stimulating VSV-ΔM51 replication with enhanced oncolysis [102]. In a recent study, Briddle and colleagues have demonstrated that the systemic administration of rhabdovirus vectors such as of VSV could effectively promote expansion of antigen specific CD8^+^ T cells. In the secondary lymphoid organ (spleen), the splenic B cells cooperated with resident DCs, which promoted downstream expansion of antigen-specific CD8^+^ T cells. This mechanism could be extrapolated in the context of using VSV not only as a primary oncolytic agent, but also as an immune booster for promoting the expansion of tumor specific T cells [103]. From these studies, it is expected that in the future, use of rVSV platforms with an immune modulator approach is likely to augment its direct oncolytic activities. 

## 8. Concluding Remarks

Long-standing historical evidence showing viruses can favorably proliferate and kill tumor cells have opened new avenues for the therapeutic use of viruses in cancer treatment. Continued interest to explore new approaches and bring novel candidate viruses as an alternate and efficacious therapy against cancer has made big strides in biomedical science. Many proof-of-concept studies have been successfully performed. In this direction, the potential of Rhabdovirus as an anticancer agent has opened the field and made VSV a formidable OV candidate. Each passing year has witnessed the creation of recombinant VSVs with guided modification targeting specific aspects of tumor lysis. More importantly, we have now learned the very fundamental aspects of virus biology in the context of cancer treatment in minute detail. AsVSV-IFNβ has entered several clinical studies; it will be exciting to see the outcome in cancer treatment. For rVSVs entering mainstream oncotherapy, the future looks more optimistic than ever; yet, many hidden hurdles that may come on the way need to be conquered. We certainly are hopeful and predict that the combinatorial approaches including immunotherapy along with virotherapy, will make an impact in future cancer therapy.

## Figures and Tables

**Table 1:** List of potential recombinant vesicular stomatitis viruses with created for oncotherapy application.

VSV Modification	Virus Description	Reference
VSV-IL4	rVSV expressing IL-4 cytokine with enhanced oncolytic activity	[81]
VSV-IFNβ	rVSV expressing IFN-β gene, show oncolytic activity against metastatic lung disease, and able to generate T cell response	[93]
VSV-IL12	rVSV is expressing murine IL-12 gene show oncolytic activity against squamous cell carcinoma.	[88]
rVSV-gG	rVSV expressing equine herpes virus-1 glycoprotein G, which acts as a broad-spectrum viral chemokine binding protein	[104]
rVSV-UL141	rVSV expressing a protein from human cytomegalovirus which down regulates the natural killer (NK) cell-activating ligand CD155 and inhibits the function of NK cell	[77]
rVSV(MΔ51)-M3	rVSV expressing the murine gammaherpesvirus-68 chemokine-binding protein M3 in modified matrix protein backbone with enhanced tumor necrosis	[78]
ΔM51-VSV	ΔM51-VSV infection activated DCs to produce proinflammatory cytokines (IL-12 and IFNs)	[105]
VSV-CD40L	rVSV expressing CD40L, a member of the TNF family expressed on the surface of activated Th cells.	[106]
VSV-p14	rVSV expressing p14 FAST protein increase oncolytic property	[80]
VSV-CD133	rVSV expressing CD133 (a marker for cancer stem cells) increase specificity for CD133 expressing tumours.	[75]
VSV-IL15	rVSV expressing secreted version of human interleukin15, it enhances both NK cell and T cell response	[60]
VSV-IL28	rVSV expressing IL-28, a member of type 3 IFN	[92]
VSV-rFlt3L	rVSV expressing the Fms-like tyrosine kinase 3 ligand (rFlt3L). rFlt3L is a growth factor which promotes the differentiation and proliferation of DC.	[107]
VSV-IFNγ	rVSV expressing IFNγ which slows tumor growth	[108]
VSV-mIFNβ-NIS	rVSV expressing IFNβ and the NIS reporter, in the presence of anti-PD-L1 antibody, it shows higher anti tumor activity	[109]
**VSV expressing suicide gene**		
VSV-TK	rVSV expressing thymidine kinase of herpes virus, increase oncolytic property	[81]
VSV(ΔM51) NIS	rVSV expressing human *NIS* gene, shows specific oncolytic activity against myeloma.	[110]
VSV-C:U	rVSV expressing the fusion suicide gene *Escherichia coli* cytosine deaminase (CD)/uracil phosphoribosyltransferase (UPRT), catalyzing the modification of 5-fluorocytosine into chemotherapeutic 5-fluorouracil	[83]
VSV-mp53 and VSV-ΔM-mp53	VSV-mp53 and VSV-ΔM-mp53 both expressing high level of functional p53 in respective backbone VSV with chemical compounds	[74]
LCL161 and VSV-ΔM51	SMC and OV therapies combination also synergize in vivo by promoting anticancer immunity through an increase in CD8^+^ T-cell response	[111]
Curcumin and VSV	Cumulative decrease in the expression of the anti-apoptotic protein, Bcl-XL, and in the phosphorylation of NF-κB and increase in the number of virus infected cells	[112]
Ruxolitinib and Polycation with VSV	Ruxolitinib and polycation improve VSV attachment and replication in HPAF-II cells	[113]
SFN (antioxidant compound sulforaphane) and VSV	SFN enhances VSVΔ51 spread in oncolytic virus-resistant cancer cells	[102]

TNF: tumor necrosis factor; DC: dendritic cells; NIS: sodium iodide symporter; SMC: Second mitochondrial activator of caspase (Smac)-mimetic compounds; NF-κB: nuclear factor kappa-light-chain-enhancer of activated B cells.

## References

[1] Kaufman H.L., Kohlhapp F.J., Zloza A. (2015). Oncolytic viruses: A new class of immunotherapy drugs. Nat. Rev. Drug Discov..

[2] Russell S.J., Peng K.W. (2007). Viruses as anticancer drugs. Trends Pharmacol. Sci..

[3] Kirn D., Martuza R.L., Zwiebel J. (2001). Replication-selective virotherapy for cancer: Biological principles, risk management and future directions. Nat. Med..

[4] Pelner L., Fowler G.A., Nauts H.C. (1958). Effect of Concurrent Infections and Their Toxins on the Course of Leukemia. Acta Med. Scand..

[5] Gross S. (1971). Measles and leukaemia. Lancet.

[6] Kelly E., Russell S.J. (2007). History of Oncolytic Viruses: Genesis to Genetic Engineering. Mol. Ther..

[7] Still G.F. (1897). On a form of chronic joint disease in children. Med.-Chir. Trans..

[8] Sorenson A.W., Street J.C. (1981). The need for comprehensive diet studies to assess the relation of lipids to cancer. Cancer Res..

[9] Fukuhara H., Homma Y., Todo T. (2010). Oncolytic virus therapy for prostate cancer. Int. J. Urol..

[10] Zhu Z., Gorman M.J., McKenzie L.D., Chai J.N., Hubert C.G., Prager B.C., Fernandez E., Richner J.M., Zhang R., Shan C. (2017). Zika virus has oncolytic activity against glioblastoma stem cells. J. Exp. Med..

[11] Betancourt D., Ramos J.C., Barber G.N. (2015). Retargeting Oncolytic Vesicular Stomatitis Virus to Human T-Cell Lymphotropic Virus Type 1-Associated Adult T-Cell Leukemia. J. Virol..

[12] Banerjee A.K. (1987). The transcription complex of vesicular stomatitis virus. Cell.

[13] Ahmed M., Lyles D.S. (1998). Effect of vesicular stomatitis virus matrix protein on transcription directed by host RNA polymerases I, II, and III. J. Virol..

[14] Enninga J., Levy D.E., Blobel G., Fontoura B.M.A. (2002). Role of nucleoporin induction in releasing an mRNA nuclear export block. Science.

[15] Her L.S., Lund E., Dahlberg J.E. (1997). Inhibition of Ran guanosine triphosphatase-dependent nuclear transport by the matrix protein of vesicular stomatitis virus. Science.

[16] Faria P.A., Chakraborty P., Levay A., Barber G.N., Ezelle H.J., Enninga J., Arana C., van Deursen J., Fontoura B.M.A. (2005). VSV disrupts the Rae1/mrnp41 mRNA nuclear export pathway. Mol. Cell.

[17] Rajani K.R., Pettit Kneller E.L., McKenzie M.O., Horita D.A., Chou J.W., Lyles D.S. (2012). Complexes of Vesicular Stomatitis Virus Matrix Protein with Host Rae1 and Nup98 Involved in Inhibition of Host Transcription. PLoS Pathog..

[18] Lee A.S.-Y., Burdeinick-Kerr R., Whelan S.P.J. (2013). A ribosome-specialized translation initiation pathway is required for cap-dependent translation of vesicular stomatitis virus mRNAs. Proc. Natl. Acad. Sci. USA.

[19] Whitlow Z.W., Connor J.H., Lyles D.S. (2006). Preferential translation of vesicular stomatitis virus mRNAs is conferred by transcription from the viral genome. J. Virol..

[20] Black B.L., Brewer G., Lyles D.S. (1994). Effect of vesicular stomatitis virus matrix protein on host-directed translation in vivo. J. Virol..

[21] Koyama A.H. (1995). Induction of apoptotic DNA fragmentation by the infection of vesicular stomatitis virus. Virus Res..

[22] Black B.L., Lyles D.S., Carolina N. (1992). Vesicular Stomatitis Virus Matrix Protein Inhibits Host Cell-Directed Transcription of Target Genes In Vivo. J. Virol..

[23] Blondel D., Harmison G.G., Schubert M. (1990). Role of matrix protein in cytopathogenesis of vesicular stomatitis virus. J. Virol..

[24] Gaddy D.F., Lyles D.S. (2005). Vesicular stomatitis viruses expressing wild-type or mutant M proteins activate apoptosis through distinct pathways. J. Virol..

[25] Balachandran S., Roberts P.C., Kipperman T., Bhalla K.N., Compans R.W., Archer D.R., Barber G.N. (2000). Alpha/beta interferons potentiate virus-induced apoptosis through activation of the FADD/Caspase-8 death signaling pathway. J. Virol..

[26] Stojdl D.F., Lichty B.D., TenOever B.R., Paterson J.M., Power A.T., Knowles S., Marius R., Reynard J., Poliquin L., Atkins H. (2003). VSV strains with defects in their ability to shutdown innate immunity are potent systemic anti-cancer agents. Cancer Cell.

[27] Gaddy D.F., Lyles D.S. (2007). Oncolytic Vesicular Stomatitis Virus Induces Apoptosis via Signaling. J. Virol..

[28] Schache P., Gürlevik E., Strüver N., Woller N., Malek N., Zender L., Manns M., Wirth T., Kühnel F., Kubicka S. (2009). VSV virotherapy improves chemotherapy by triggering apoptosis due to proteasomal degradation of Mcl-1. Gene Ther..

[29] Qi X., Du L., Chen X., Chen L., Yi T., Chen X., Wen Y., Wei Y., Zhao X. (2016). VEGF-D-enhanced lymph node metastasis of ovarian cancer is reversed by vesicular stomatitis virus matrix protein. Int. J. Oncol..

[30] Mahoney D.J., Lefebvre C., Allan K., Brun J., Sanaei C.A., Baird S., Pearce N., Grönberg S., Wilson B., Prakesh M. (2011). Virus-Tumor Interactome Screen Reveals ER Stress Response Can Reprogram Resistant Cancers for Oncolytic Virus-Triggered Caspase-2 Cell Death. Cancer Cell.

[31] Rosebeck S., Sudini K., Chen T., Leaman D.W. (2011). Involvement of Noxa in mediating cellular ER stress responses to lytic virus infection. Virology.

[32] Breitbach C.J., Paterson J.M., Lemay C.G., Falls T.J., McGuire A., Parato K.A., Stojdl D.F., Daneshmand M., Speth K., Kirn D. (2007). Targeted Inflammation During Oncolytic Virus Therapy Severely Compromises Tumor Blood Flow. Mol. Ther..

[33] Breitbach C.J., Silva N.S., de Falls T.J., Aladl U., Evgin L., Paterson J., Sun Y.Y., Roy D.G., Rintoul J.L., Daneshmand M. (2011). Targeting Tumor Vasculature With an Oncolytic Virus. Mol. Ther..

[34] Brahimi-Horn C., Berra E., Pouysségur J. (2001). Hypoxia: The tumor’s gateway to progression along the angiogenic pathway. Trends Cell Biol..

[35] Probst G., Riedinger H.J., Martin P., Engelcke M., Probst H. (1999). Fast control of DNA replication in response to hypoxia and to inhibited protein synthesis in CCRF-CEM and HeLa cells. Biol. Chem..

[36] Brown J.M. (2002). Tumor microenvironment and the response to anticancer therapy. Cancer Biol. Ther..

[37] Connor J.H., Naczki C., Koumenis C., Lyles D.S. (2004). Replication and Cytopathic Effect of Oncolytic Vesicular Stomatitis Virus in Hypoxic Tumor Cells In Vitro and In Vivo. J. Virol..

[38] Singh S.R., Walters K.F., Port G.R. (2001). Behaviour of the adult seven spot ladybird, *Coccinella septempunctata* (Coleoptera: *Coccinellidae*), in response to dimethoate residue on bean plants in the laboratory. Bull. Entomol. Res..

[39] Stark G.R., Kerr I.M., Williams B.R., Silverman R.H., Schreiber R.D. (1998). How cells respond to interferons. Annu. Rev. Biochem..

[40] Balachandran S., Barber G.N. (2000). Vesicular Stomatitis Virus (VSV) Therapy of Tumors. IUBMB Life.

[41] D’agostino P.M., Amenta J.J., Reiss C.S. (2009). IFN-β-induced alteration of VSV protein phosphorylation in neuronal cells. Viral Immunol..

[42] Stojdl D., Lichty B., Knowles S., Marius R., Atkins H., Sonenberg N., Bell J.C. (2000). Exploiting tumor-specific defects in the interferon pathway with a previously unknown oncolytic virus. Nat. Med..

[43] Zhang K.X., Matsui Y., Hadaschik B.A., Lee C., Jia W., Bell J.C., Fazli L., So A.I., Rennie P.S. (2010). Down-regulation of type I interferon receptor sensitizes bladder cancer cells to vesicular stomatitis virus-induced cell death. Int. J. Cancer.

[44] Westcott M.M., Liu J., Rajani K., D’Agostino R., Lyles D.S., Porosnicu M. (2015). Interferon β and Interferon α2a Differentially Protect Head and Neck Cancer Cells from Vesicular Stomatitis Virus-Induced Oncolysis. J. Virol..

[45] Balachandran S., Porosnicu M., Barber G.N. (2001). Oncolytic activity of vesicular stomatitis virus is effective against tumors exhibiting aberrant p53, Ras, or myc function and involves the induction of apoptosis. J. Virol..

[46] Noser J.A., Mael A.A., Sakuma R., Ohmine S., Marcato P., WK Lee P., Ikeda Y. (2007). The RAS/Raf1/MEK/ERK Signaling Pathway Facilitates VSV-mediated Oncolysis: Implication for the Defective Interferon Response in Cancer Cells. Mol. Ther..

[47] Burns J.C., Friedmann T., Drievert W., Burrascano M., Yee J.-K. (1993). Vesicular stomatitis virus G glycoprotein pseudotyped retroviral vectors: Concentration to very high titer and efficient gene transfer into mammalian and nonmammalian cells (gene therapy/zebrafish). Genetics.

[48] Van den Pol A.N., Dalton K.P., Rose J.K. (2002). Relative neurotropism of a recombinant rhabdovirus expressing a green fluorescent envelope glycoprotein. J. Virol..

[49] Kelly E.J., Nace R., Barber G.N., Russell S.J. (2010). Attenuation of Vesicular Stomatitis Virus Encephalitis through MicroRNA Targeting. J. Virol..

[50] Takamizawa J., Konishi H., Yanagisawa K., Tomida S., Osada H., Endoh H., Harano T., Yatabe Y., Nagino M., Nimura Y. (2004). Reduced Expression of the let-7 MicroRNAs in Human Lung Cancers in Association with Shortened Postoperative Survival Advances in Brief Reduced Expression of the let-7 MicroRNAs in Human Lung Cancers in Association with Shortened Postoperative Survival. Cancer Res..

[51] Edge R.E., Falls T.J., Brown C.W., Lichty B.D., Atkins H., Bell J.C. (2008). A let-7 MicroRNA-sensitive Vesicular Stomatitis Virus Demonstrates Tumor-specific Replication. Mol. Ther..

[52] Lawson N.D., Stillman E.A., Whitt M.A., Rose J.K. (1995). Recombinant vesicular stomatitis viruses from DNA. Proc. Natl. Acad. Sci. USA.

[53] Publicover J., Ramsburg E., Robek M., Rose J.K. (2006). Rapid Pathogenesis Induced by a Vesicular Stomatitis Virus Matrix Protein Mutant: Viral Pathogenesis Is Linked to Induction of Tumor Necrosis Factor α. J. Virol..

[54] Ammayappan A., Nace R., Peng K.-W., Russell S.J. (2013). Neuroattenuation of Vesicular Stomatitis Virus through Picornaviral Internal Ribosome Entry Sites. J. Virol..

[55] Janelle V., Brassard F., Lapierre P., Lamarre A., Poliquin L. (2011). Mutations in the Glycoprotein of Vesicular Stomatitis Virus Affect Cytopathogenicity: Potential for Oncolytic Virotherapy. J. Virol..

[56] Publicover J., Ramsburg E., Rose J.K. (2004). Characterization of nonpathogenic, live, viral vaccine vectors inducing potent cellular immune responses. J. Virol..

[57] Duntsch C.D., Zhou Q., Jayakar H.R., Weimar J.D., Robertson J.H., Pfeffer L.M., Wang L., Xiang Z., Whitt M.A. (2004). Recombinant vesicular stomatitis virus vectors as oncolytic agents in the treatment of high-grade gliomas in an organotypic brain tissue slice-glioma coculture model. J. Neurosurg..

[58] Ahmed M., Puckett S., Lyles D.S. (2010). Susceptibility of breast cancer cells to an oncolytic matrix (M) protein mutant of vesicular stomatitis virus. Cancer Gene Ther..

[59] Muik A., Stubbert L.J., Jahedi R.Z., Geib Y., Kimpel J., Dold C., Tober R., Volk A., Klein S., Dietrich U. (2014). Re-engineering vesicular stomatitis virus to abrogate neurotoxicity, circumvent humoral immunity, and enhance oncolytic potency. Cancer Res..

[60] Stephenson K.B., Barra N.G., Davies E., Ashkar A.A., Lichty B.D. (2012). Expressing human interleukin-15 from oncolytic vesicular stomatitis virus improves survival in a murine metastatic colon adenocarcinoma model through the enhancement of anti-tumor immunity. Cancer Gene Ther..

[61] Tesfay M.Z., Kirk A.C., Hadac E.M., Griesmann G.E., Federspiel M.J., Barber G.N., Henry S.M., Peng K., Russell J. (2013). PEGylation of Vesicular Stomatitis Virus Extends Virus Persistence in Blood Circulation of Passively Immunized Mice. J. Virol..

[62] Muik A., Dold C., Geiß Y., Volk A., Werbizki M., Dietrich U., von Laer D. (2012). Semireplication-competent vesicular stomatitis virus as a novel platform for oncolytic virotherapy. J. Mol. Med..

[63] Van den Pol A.N., Davis J.N. (2013). Highly Attenuated Recombinant Vesicular Stomatitis Virus VSV-12’GFP Displays Immunogenic and Oncolytic Activity. J. Virol..

[64] Wollmann G., Rogulin V., Simon I., Rose J.K., van den Pol A.N. (2010). Some Attenuated Variants of Vesicular Stomatitis Virus Show Enhanced Oncolytic Activity against Human Glioblastoma Cells relative to Normal Brain Cells. J. Virol..

[65] Dreja H., Piechaczyk M. (2006). The effects of N-terminal insertion into VSV-G of an scFv peptide. Virol. J..

[66] Guibinga G.H., Hall F.L., Gordon E.M., Ruoslahti E., Friedmann T. (2004). Ligand-modified vesicular stomatitis virus glycoprotein displays a temperature-sensitive intracellular trafficking and virus assembly phenotype. Mol. Ther..

[67] Ammayappan A., Peng K.-W., Russell S.J. (2013). Characteristics of oncolytic vesicular stomatitis virus displaying tumor-targeting ligands. J. Virol..

[68] Desgrosellier J.S., Cheresh D. (2010). Integrins in cancer: Biological implications and therapeutic opportunities. Nat. Rev. Cancer.

[69] Padmashali R.M., Andreadis S.T. (2011). Engineering fibrinogen-binding VSV-G envelope for spatially- and cell-controlled lentivirus delivery through fibrin hydrogels. Biomaterials.

[70] Bergman I., Whitaker-dowling P., Gao Y., Griffin J.A., Watkins S.C. (2003). Vesicular stomatitis virus expressing a chimeric Sindbis glycoprotein containing an Fc antibody binding domain targets to Her2/neu overexpressing breast cancer cells. Virology.

[71] Gao Y., Whitaker-Dowling P., Griffin J.A., Barmada M.A., Bergman I. (2009). Recombinant vesicular stomatitis virus targeted to Her2/neu combined with anti-CTLA4 antibody eliminates implanted mammary tumors. Cancer Gene Ther..

[72] Gao Y., Whitaker-Dowling P., Griffin J.A., Bergman I. (2012). Treatment with targeted vesicular stomatitis virus generates therapeutic multifunctional anti-tumor memory CD4 T cells. Cancer Gene Ther..

[73] Kannan K., Amariglio N., Rechavi G., Jakob-Hirsch J., Kela I., Kaminski N., Getz G., Domany E., Givol D. (2001). DNA microarrays identification of primary and secondary target genes regulated by p53. Oncogene.

[74] Heiber J.F., Barber G.N. (2011). Vesicular Stomatitis Virus Expressing Tumor Suppressor p53 Is a Highly Attenuated, Potent Oncolytic Agent. J. Virol..

[75] Nakamura T., Peng K.-W., Vongpunsawad S., Harvey M., Mizuguchi H., Hayakawa T., Cattaneo R., Russell S.J. (2004). Antibody-targeted cell fusion. Nat. Biotechnol..

[76] Kleinlützum D., Hanauer J.D.S., Muik A., Hanschmann K.-M., Kays S.-K., Ayala-Breton C., Peng K.-W., Mühlebach M.D., Abel T., Buchholz C.J. (2017). Enhancing the Oncolytic Activity of CD133-Targeted Measles Virus: Receptor Extension or Chimerism with Vesicular Stomatitis Virus Are Most Effective. Front. Oncol..

[77] Altomonte J., Wu L., Meseck M., Chen L., Ebert O., Garcia-Sastre A., Fallon J., Mandeli J., Woo S.L.C. (2009). Enhanced Oncolytic Potency of Vesicular Stomatitis Virus via Vector-Mediated Inhibition of NK and NKT Cells. Cancer Gene Ther..

[78] Wu L., Huang T., Meseck M., Altomonte J., Ebert O., Shinozaki K., García-Sastre A., Fallon J., Mandeli J., Woo S.L.C. (2008). rVSV(M Delta 51)-M3 is an effective and safe oncolytic virus for cancer therapy. Hum. Gene Ther..

[79] Ebert O., Shinozaki K., Kournioti C., Ebert O., Shinozaki K., Kournioti C., Park M., Garcı A. (2004). Syncytia Induction Enhances the Oncolytic Potential of Vesicular Stomatitis Virus in Virotherapy for Cancer Syncytia Induction Enhances the Oncolytic Potential of Vesicular Stomatitis Virus in Virotherapy for Cancer. Cancer Res..

[80] Le Boeuf F., Gebremeskel S., McMullen N., He H., Greenshields A.L., Hoskin D.W., Bell J.C., Johnston B., Pan C., Duncan R. (2017). Reovirus FAST Protein Enhances Vesicular Stomatitis Virus Oncolytic Virotherapy in Primary and Metastatic Tumor Models. Mol. Ther. Oncolytics.

[81] Fernandez M., Porosnicu M., Markovic D., Barber G.N. (2002). Genetically engineered vesicular stomatitis virus in gene therapy: Application for treatment of malignant disease. J. Virol..

[82] Lal S., Lauer U.M., Niethammer D., Beck J.F., Schlegel P.G. (2000). Suicide genes: Past, present and future perspectives. Rev. Immunol. Today.

[83] Porosnicu M., Mian A., Barber G.N. (2003). The Oncolytic Effect of Recombinant Vesicular Stomatitis Virus Is Enhanced by Expression of the Fusion Cytosine Deaminase/Uracil Phosphoribosyltransferase Suicide Gene. Cancer Res..

[84] Leveille S., Samuel S., Goulet M., Hiscott J. (2011). Enhancing VSV oncolytic activity with an improved cytosine deaminase suicide gene strategy. Cancer Gene Ther..

[85] Miest T.S., Cattaneo R. (2013). New viruses for cancer therapy: Meeting clinical needs. Nat. Rev. Microbiol..

[86] Benedetti S., Pirola B., Pollo B., Magrassi L., Bruzzone M.G., Rigamonti D., Galli R., Selleri S., di Meco F., de Fraja C. (2000). Gene therapy of experimental brain tumors using neural progenitor cells. Nat. Med..

[87] Tepper R.I., Pattengale P.K., Leder P. (1989). Murine interleukin-4 displays potent anti-tumor activity in vivo. Cell.

[88] Shin E.J., Wanna G.B., Choi B., Iii D.A., Ebert O., Genden E.M., Woo S.L. (2007). Interleukin-12 Expression Enhances Vesicular Stomatitis Virus Oncolytic Therapy in Murine Squamous Cell Carcinoma. Laryngoscope.

[89] Ramsburg E., Publicover J., Buonocore L., Poholek A., Robek M., Palin A., John K., Rose J.K. (2005). A Vesicular Stomatitis Virus Recombinant Expressing Granulocyte-Macrophage Colony-Stimulating Factor Induces Enhanced T-Cell Responses and Is Highly Attenuated for Replication in Animals A Vesicular Stomatitis Virus Recombinant Expressing Granulocyte-Macr. J. Virol..

[90] Bergman I., Griffin J.A., Gao Y., Whitaker-dowling P. (2007). Treatment of implanted mammary tumors with recombinant vesicular stomatitis virus targeted to Her2/neu. Int. J. Cancer.

[91] Miller J., Bidula S.M., Jensen T.M., Reiss C.S. (2009). Cytokine-modified VSV is attenuated for neural pathology, but is both highly immunogenic and oncolytic. Int. J. Interferon Cytokine Mediat. Res..

[92] Wongthida P., Diaz R.M., Galivo F., Kottke T., Thompson J., Pulido J., Pavelko K., Pease L., Melcher A., Vile R. (2010). Type III IFN interleukin-28 mediates the antitumor efficacy of oncolytic virus VSV in immune-competent mouse models of cancer. Cancer Res..

[93] Obuchi M., Fernandez M., Barber G.N. (2003). Development of Recombinant Vesicular Stomatitis Viruses That Exploit Defects in Host Defense To Augment Specific Oncolytic Activity. J. Virol..

[94] Velazquez-Salinas L., Naik S., Pauszek S.J., Peng K.-W., Russell S.J., Rodriguez L.L. (2017). Oncolytic Recombinant Vesicular Stomatitis Virus (VSV) Is Nonpathogenic and Nontransmissible in Pigs, a Natural Host of VSV. Hum. Gene Ther. Clin. Dev..

[95] Zhang L., Steele M.B., Jenks N., Grell J., Suksanpaisan L., Naik S., Federspiel M.J., Lacy M.Q., Russell S.J., Peng K.-W. (2016). Safety Studies in Tumor and Non-Tumor-Bearing Mice in Support of Clinical Trials Using Oncolytic VSV-IFNβ-NIS. Hum. Gene Ther. Clin. Dev..

[96] Naik S., Galyon G.D., Jenks N.J., Steele M.B., Miller A.C., Allstadt S.D., Suksanpaisan L., Peng K.W., Federspiel M.J., Russell S.J. (2018). Comparative Oncology Evaluation of Intravenous Recombinant Oncolytic Vesicular Stomatitis Virus Therapy in Spontaneous Canine Cancer. Mol. Cancer Ther..

[97] Galivo F., Diaz R.M., Thanarajasingam U., Jevremovic D., Wongthida P., Thompson J., Kottke T., Barber G.N., Melcher A., Vile R.G. (2010). Interference of CD40L-mediated tumor immunotherapy by oncolytic vesicular stomatitis virus. Hum. Gene Ther..

[98] Wongthida P., Diaz R.M., Galivo F., Kottke T., Thompson J., Melcher A., Vile R. (2011). VSV Oncolytic Virotherapy in the B16 Model Depends Upon Intact MyD88 Signaling. Mol. Ther..

[99] Barber G.N. (2005). VSV-tumor selective replication and protein translation. Oncogene.

[100] Power A.T., Wang J., Falls T.J., Paterson J.M., Parato K.A., Lichty B.D., Stojdl D.F., Forsyth P.A.J., Atkins H., Bell J.C. (2007). Carrier cell-based delivery of an oncolytic virus circumvents antiviral immunity. Mol. Ther..

[101] Diaz R.M., Galivo F., Kottke T., Wongthida P., Qiao J., Thompson J., Valdes M., Barber G., Vile R.G. (2007). Oncolytic Immunovirotherapy for Melanoma Using Vesicular Stomatitis Virus. Cancer Res..

[102] Olagnier D., Lababidi R.R., Hadj S.B., Sze A., Liu Y., Naidu S.D., Ferrari M., Jiang Y., Chiang C., Beljanski V. (2017). Activation of Nrf2 Signaling Augments Vesicular Stomatitis Virus Oncolysis via Autophagy-Driven Suppression of Antiviral Immunity. Mol. Ther..

[103] Bridle B.W., Nguyen A., Salem O., Zhang L., Koshy S., Clouthier D., Chen L., Pol J., Swift S.L., Bowdish D.M.E. (2016). Privileged Antigen Presentation in Splenic B Cell Follicles Maximizes T Cell Responses in Prime-Boost Vaccination. J. Immunol..

[104] Altomonte J., Wu L., Chen L., Meseck M., Ebert O., Garcia-Sastre A., Fallon J., Woo S.L. (2008). Exponential enhancement of oncolytic vesicular stomatitis virus potency by vector-mediated suppression of inflammatory responses in vivo. Mol. Ther..

[105] Boudreau J.E., Bridle B.W., Stephenson K.B., Jenkins K.M., Brunellière J., Bramson J.L., Lichty B.D., Wan Y. (2009). Recombinant Vesicular Stomatitis Virus Transduction of Dendritic Cells Enhances Their Ability to Prime Innate and Adaptive Antitumor Immunity. Mol. Ther..

[106] Galivo F., Diaz R.M., Wongthida P., Thompson J., Kottke T., Barber G., Melcher A., Vile R. (2009). Single-cycle viral gene expression, rather than progressive replication and oncolysis, is required for VSV therapy of B16 melanoma. Gene Ther..

[107] Leveille S., Goulet M.L., Lichty B.D., Hiscott J. (2011). Vesicular stomatitis virus oncolytic treatment interferes with tumor-associated dendritic cell functions and abrogates tumor antigen presentation. J. Virol..

[108] Bourgeois-Daigneault M.-C., Roy D.G., Falls T., Twumasi-Boateng K., St-Germain L.E., Marguerie M., Garcia V., Selman M., Jennings V.A., Pettigrew J. (2016). Oncolytic vesicular stomatitis virus expressing interferon-γ has enhanced therapeutic activity. Mol. Ther. Oncol..

[109] Shen W., Patnaik M.M., Ruiz A., Russell S.J., Peng K.W. (2016). Immunovirotherapy with vesicular stomatitis virus and PD-L1 blockade enhances therapeutic outcome in murine acute myeloid leukemia. Blood.

[110] Goel A., Carlson S.K., Classic K.L., Greiner S., Naik S., Power A.T., Bell J.C., Russell S.J. (2007). Radioiodide imaging and radiovirotherapy of multiple myeloma using VSV(Delta51)-NIS, an attenuated vesicular stomatitis virus encoding the sodium iodide symporter gene. Blood.

[111] Kim D.S., Dastidar H., Zhang C., Zemp F.J., Lau K., Ernst M., Rakic A., Sikdar S., Rajwani J., Naumenko V. (2017). Smac mimetics and oncolytic viruses synergize in driving anticancer T-cell responses through complementary mechanisms. Nat. Commun..

[112] Fehl D.J., Ahmed M. (2017). Curcumin promotes the oncoltyic capacity of vesicular stomatitis virus for the treatment of prostate cancers. Virus Res..

[113] Felt S.A., Droby G.N., Grdzelishvili V.Z. (2017). Ruxolitinib and Polycation Combination Treatment Overcomes Multiple Mechanisms of Resistance of Pancreatic Cancer Cells to Oncolytic Vesicular Stomatitis Virus. J. Virol..

